# Impact of Different Approaches to Kidney Transplant with and without Chronic Hemodialysis on Cardiac Function and Morphology: A Case–Control Study

**DOI:** 10.3390/jcm10173913

**Published:** 2021-08-30

**Authors:** Marta Obremska, Dorota Kamińska, Magdalena Krawczyk, Magdalena Krajewska, Wojciech Kosmala

**Affiliations:** 1 Department of Cardiovascular Imaging, Institute of Heart Diseases, Wroclaw Medical University, 50-556 Wroclaw, Borowska 213, Poland; krawmag@gmail.com (M.K.); wojciech.kosmala@umed.wroc.pl (W.K.); 2 Department of Nephrology and Transplantation Medicine, Wroclaw Medical University, 50-556 Wroclaw, Borowska 213, Poland; dorota.kaminska@umed.wroc.pl (D.K.); magdalena.krajewska@umed.wroc.pl (M.K.)

**Keywords:** cardiac function, cardiac morphology, chronic hemodialysis, preemptive kidney transplant, end-stage renal disease

## Abstract

Patients with end-stage renal disease have higher cardiovascular morbidity and mortality compared with the general population. Preemptive kidney transplant (KTx) has been shown to be associated with improved survival, better quality of life, lower healthcare burden, and reduced cardiovascular risk. In this case–control study, we investigated the cardiovascular benefits of two approaches to KTx: with and without previous chronic hemodialysis. We enrolled 21 patients who underwent preemptive KTx and 21 matched controls who received chronic hemodialysis before KTx. Cardiac morphological and functional parameters were assessed by echocardiography. Overall, patients undergoing preemptive KTx showed less extensive cardiac damage compared with controls, as evidenced by higher global longitudinal strain, peak atrial and contractile strain, and early diastolic mitral annular velocity as well as a lower left ventricular mass, left atrial volume index, and the ratio of mitral inflow early diastolic velocity to the mitral annular early diastolic velocity. In the multivariable analysis, the presence of chronic hemodialysis prior to KTx was an independent determinant of post-transplant cardiac functional and structural remodeling. These findings may have important clinical implications, supporting the use of preemptive KTx as a preferred treatment strategy in patients with end-stage renal disease.

## 1. Introduction

Cardiovascular morbidity and mortality in patients with end-stage renal diseases is higher than in the general population [1,2]. Preemptive kidney transplantation (KTx), defined as a transplant prior to hemodialysis (HD), has been demonstrated to provide higher rates of survival, better quality of life, and healthcare costs benefits [3]. Post-transplant cardiovascular risk has been found to be significantly lower in patients undergoing preemptive KTx, irrespective of the earlier cardiovascular disease [3,4]. Among the possible reasons behind the improved mortality in patients receiving preemptive KTx is a reduced rate of cardiovascular complications attributable to the avoidance of pre-transplant HD [4,5,6].

Apart from vascular factors associated with accelerated atherosclerosis and arterial calcification, myocardial impairment has been postulated to deteriorate the clinical course in KTx recipients subjected to previous HD treatment [7,8]. Evidence exists that left ventricular (LV) hypertrophy and functional (systolic and diastolic) abnormalities contribute to the development heart failure—a clinical manifestation of uremic cardiomyopathy [9]. HD as a kind of renal replacement therapy alleviates the major consequences of renal failure; however, its potential role in aggravating cardiac structural and functional derangements in KTx recipients is underexplored. Despite the increasing interest in preemptive KTx in recent years, the plausible benefits from this option for the myocardium have not yet been documented.

Therefore, in this study, we sought to investigate whether the treatment strategy based on preemptive KTx is associated with a better post-transplant cardiac functional and morphological profile than the approach including chronic dialysis before KTx.

## 2. Materials and Methods

### 2.1. Study Design

The databases of local transplant outpatient clinics as well as a registry of preemptive KTx were searched to identify patients who underwent preemptive KTx. Preemptive KTx was defined as a transplant before starting chronic hemodialysis. The inclusion criteria were first KTx, age above 18 years, and consent to participate in the study. The exclusion criteria were subsequent KTx, history of atrial fibrillation, moderate or severe valvular heart disease, ischemic heart disease, and poor-quality echocardiographic images. Finally, 21 patients were enrolled in the study group. These subjects were individually matched on the basis of sex, age (±6 years), and time after KTx (< ±30%) with 21 patients who had undergone at least a 12-month HD before KTx (a control group) and satisfied the inclusion and exclusion criteria. All enrollees from the study and control group had been placed on the transplant waiting list after achieving a steady eGFR of less than 15 mL/min/1.73 m^2^.

Echocardiography was performed during the patient’s routine appointment at the transplant outpatient clinic. The time after KTx refers to the date of the echocardiographic examination. Likewise, on the day of echocardiography, routine laboratory assessments including of serum creatinine, eGFR, and hemoglobin were performed. Information on immunosuppressive and antihypertensive medications use and comorbidities such as hypertension, diabetes, and ischemic heart disease was also obtained. Ischemic heart disease was excluded during the process of pre-transplant evaluation on the basis of negative history and negative stress echocardiography. Coronary angiography was performed only in two patients from the control group, showing no significant atherosclerotic lesions. No participant reported symptoms of angina. The presence and localization of functional arteriovenous fistula (AVF) was verified in each patient.

Investigations were in accordance with the Declaration of Helsinki and were approved by the Local Bioethical Committee (No 170/2021). Informed written consent was obtained from each study participant.

### 2.2. Echocardiography

Echocardiographic imaging was performed using standard equipment (Vivid e9, GE Medical Systems, Horten, Norway) with a M5S phased array multifrequency transducer. Imaging data were analyzed offline after being saved in the digital format on a secure server.

### 2.3. Conventional and Tissue Doppler Imaging

The cardiac dimensions and wall thicknesses were measured according to recommendations of the American Society of Echocardiography and the European Association of Cardiovascular Imaging [10]. The LV volumes and ejection fraction were assessed using a modified Simpson’s method, whereas the left atrial (LA) volumes were assessed using the area–length method. The LV inflow parameters including peak early (E) and late diastolic flow velocity (A), and deceleration time of early diastolic flow wave (DT) were measured from the apical four-chamber view by pulsed-wave Doppler with the sample volume placed between the tips of the mitral leaflets. A pulsed-wave tissue Doppler was used to evaluate the peak early diastolic tissue velocity (e’) at the septal and lateral aspects of the mitral annulus. The ratio of mitral inflow’s early diastolic velocity to the average e’ velocity from both parts of the mitral annulus (E/e’) was calculated to approximate the LV filling pressure.

### 2.4. Speckle-Tracking Imaging

Left ventricular longitudinal deformation was evaluated by semi-automated two-dimensional speckle tracking (Echopac v.202; GE Medical Systems, Horten, Norway) in the three apical views at a temporal resolution of 60–90 frames/s. The average negative value on the strain curve was presented as global longitudinal strain (GLS). The apical four- and two-chamber views were used to evaluate LA longitudinal strain, and the onset of QRS was accepted as the zero-reference point. The peak atrial longitudinal strain (PALS; corresponding to LA reservoir function) was measured as the peak value of longitudinal strain during LV systole, and the peak atrial contractile strain (PACS, corresponding to atrial contractile function) was assessed as the value of strain at the onset of the P wave on electrocardiography. The final LA strain values were calculated as the average of both apical views. Right ventricular (RV) GLS (RVGLS) was assessed from the RV-focused apical four-chamber view and calculated as the mean of the basal, mid, and apical segments of the RV free wall. All echocardiographic parameters were averaged over three consecutive cardiac cycles and were reported as absolute values.

### 2.5. Statistical Analysis

The continuous variables with normal distribution were presented as the mean and standard deviation (SD) and compared with an unpaired Student’s t-test. The not normally distributed variables were presented as median and interquartile ranges (IQRs) and compared with the Mann–Whitney test. The discrete variables were presented as numbers and percentages and compared with the chi-squared test with the Yeates correction when indicated. The homogeneity of variances was assessed by the Levene test. The Pearson correlation coefficient for parametric variables and Spearman correlation for non-parametric variables were used to analyze the relationships between echocardiographic parameters describing myocardial structure and function, and the clinical and laboratory parameters. A series of stepwise multiple linear regression models was developed to identify the independent determinants of cardiac function and morphology parameters in renal transplant recipients. The components of these models were selected on the basis of anticipated and univariate association. The following variables were tested: age, sex, BMI, eGFR, hypertension, diabetes mellitus, pre-transplant HD, and presence of AVF. The variables were placed in the models in the order of statistical significance in the univariate analyses. All calculations were performed using standard statistical software (Statistica version 13.3, TIBCO Software Inc., Palo Alto, CA, USA). A *p*-value of less than 0.05 was regarded as statistically significant.

## 3. Results

### 3.1. Characteristics of Participants

The study group included 21 patients who underwent preemptive KTx and 21 controls who received chronic HD before KTx. Kidney transplants in both groups were performed between 2005 and 2020. The median duration of HD in the control group was 29 months (interquartile range, 12–72). In the study group, preemptive KTx was performed in nine patients as an elective procedure without vascular access for HD and with a family member as a living donor. The remaining 12 patients were put on the transplant waiting list and underwent preemptive KTx from a deceased donor while preparing for HD. Three of those patients were scheduled for regular HD with vascular access, and their AVFs remained functional throughout the study. The remaining nine patients underwent peritoneal dialysis for a maximum of three months before preemptive KTx without AVF. In the control group, 18 patients received a kidney graft from a deceased donor and three patients received a kidney graft from a living donor. A functioning AVF was found in 14 patients, while in the remaining seven participants, AVF closed spontaneously after KTx.

The demographic, clinical, and laboratory characteristics of the subsets separated according to the approach to KTx are presented in Table 1. Except for the frequency of patent AVF, no significant inter-group differences were shown.

### 3.2. Echocardiographic Parameters

**Figure 1:**
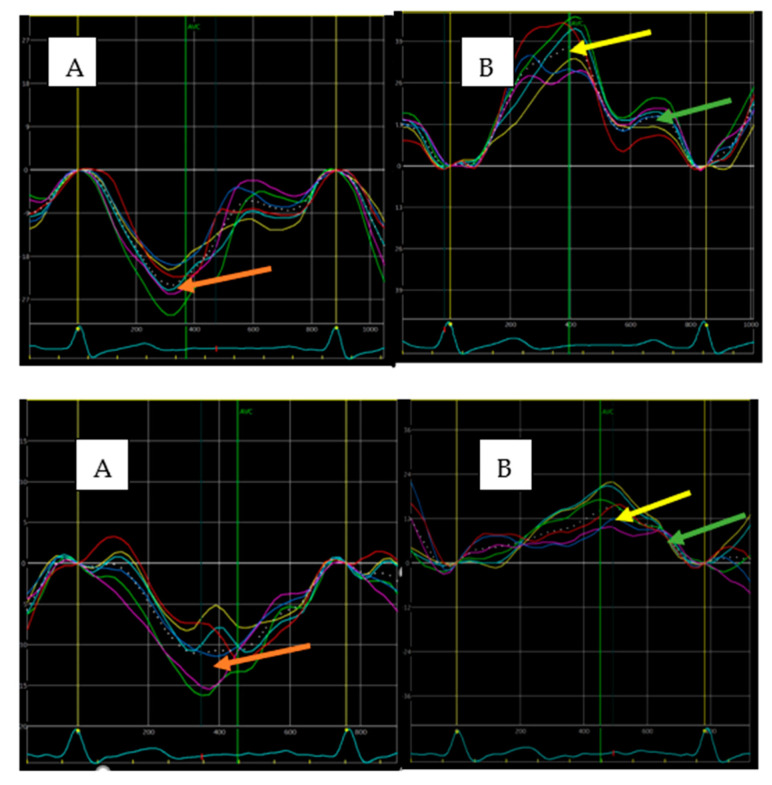
Examples of strain curves illustrating LV (**A**) and LA (**B**) function in HD (**upper** panel) and preemptive KTx patients (**lower** panel) obtained from the apical four-chamber view. Higher values of LV strain (24.8% vs. 11.3%; red arrows), PALS (36.7% vs. 15.1%; yellow arrows), and PACS (16.7% vs. 10.2%; green arrows) were found in patient from the preemptive KTx group.

In comparison with the preemptive KTx subset, patients with HD prior to KTx were characterized by a worse post-transplant cardiac morphological and functional profile, specifically larger LV mass and LA size and more impaired LV and RV longitudinal systolic function (lower GLS and RVGLS, respectively), LV diastolic function (lower e’ and higher E/e’), and LA function (lower PALS and PACT; Table 2). Examples of LV and LA strain curves in both study subgroups are presented in Figure 1.

Correlates of cardiac functional and morphological characteristics.

The univariate associations of cardiac functional and morphological parameters with demographic and clinical characteristics are presented in Table 3.

In the multivariable analysis, the independent determinants were patient age for GLS, LVMI, LAVI, and PACS; sex for GLS, LVMI, LAVI, and E/e’; hypertension for LVMI and PACS; BMI for LAVI; eGFR for LVMI, LAVI, and E/e’; and the presence of chronic HD prior to KTx for GLS, LVMI, LAVI, PALS, PACS, and E/e’ (Table 4).

## 4. Discussion

The major finding of the current study is that, in comparison with the chronic dialysis-based approach to kidney transplantation, preemptive KTx is associated with a less profound cardiac functional and structural remodeling, as evidenced by higher GLS, PALS, PACT, e’, and RVGLS and lower E/e’ ratio, LV mass, and LAVI. Thus, preemptive KTx may provide less substrates for cardiovascular disease, including heart failure, in the high risk population of renal transplant recipients.

Considerable advances in pharmacotherapy and dialysis techniques have led to increased survival among dialysis patients in recent decades, but the overall mortality rate remains high [11,12,13]. The health risks associated with dialysis are particularly heightened during the first 3 months after the initiation of this treatment. Previous studies showed longer patient and graft survival in preemptive KTx recipients [14,15,16]. The mortality benefit in preemptive KTx is multifactorial, with the absence of catheter-associated infections and vascular-access-associated complications, and the lower incidence of CV events being the major contributors.

Previous research evaluating cardiac function and morphology after KTx did not focus on preemptive transplant. To our knowledge, this is the first study to compare echocardiographic characteristics in kidney transplant recipients with and without prior chronic HD. We found that preemptive KTx was associated with a better morpho-functional profile of the left-sided heart chambers and better systolic function of the right ventricle.

Among the well-recognized markers of subclinical myocardial impairment, patients with previous chronic HD showed greater abnormalities in both LV diastolic parameters and global longitudinal deformation, indicating greater LV impairment. Furthermore, pretransplant HD was an independent determinant of posttransplant LV diastolic and longitudinal systolic functions. In the natural history of myocardial disease, abnormalities in these two LV functional domains appear very early, when LV ejection fraction is still preserved, and then parallelly progress, contributing to the development of heart failure symptoms in the later stages [17]. Both GLS and diastolic parameters, especially E/e’, are strong independent predictors of mortality and cardiovascular events in the general population and KTx recipients [18,19,20,21]. Accordingly, our findings on the differences in LV diastolic and longitudinal systolic status depending on the approach to KTx are consistent with and might partly explain the higher cardiovascular risk in patients subjected to prior dialysis treatment.

The spectrum of cardiac derangements indicative of more severe myocardial disease in the HD subset also included a higher LV mass—a well-known consequence of altered cardiac loading associated with renal disorders [22], and more profound LA remodeling reflecting both LV dysfunction as well as in situ LA disease, predisposing to the development of atrial fibrillation [23,24]. Left ventricular hypertrophy, promoting LV dysfunction and triggering ventricular arrhythmias, as well as LA abnormalities are proven prognosticators in patients with end-stage renal disease [25,26,27]. The larger accumulation of these aberrations in renal transplant recipients with prior HD may also account for worse prognosis in this KTx category.

The only RV parameter differentiating both approaches to KTx was RVGLS (lower in the HD group). This confirms the superiority of a myocardial deformation analysis over conventional echocardiographic indices [28] and provides further evidence for the greater cardiac impairment associated with pretransplant HD.

The pathophysiology behind myocardial derangements in chronic kidney disease is complex; however, especially in the context of longitudinal systolic and diastolic abnormalities, the contribution of diffuse tissue processes including interstitial fluid retention and fibrosis should be emphasized [29].

Although all patients undergoing preemptive KTx were listed for a transplant at an eGFR of less than 15 mL/min/1.73 m^2^, we cannot exclude that the possible shorter exposure to the impact of renal failure itself (i.e., beyond the effect of HD) might have contributed to less extensive cardiac damage in this subset. However, irrespective of the mechanisms of interaction with the cardiovascular system, the preemptive KTx strategy seems to cause less detriment to the myocardium than the HD-based approach. Unfortunately, because of the shortage of donors, the accessibility to this KTx modality is still limited. To better address cardiovascular problems in KTx recipients, the post-transplant reversibility of myocardial abnormalities should be more extensively explored, which might help define priorities on the KTx waiting list.

**Limitations.** Several study limitations should be acknowledged. First, the number of patients in each group was small. Second, we did not have complete datasets for between-group comparisons of pre-transplant cardiac structure and function. However, we might presume that the matching process resulted in similar cardiac profiles in both study groups. Third, the case–control design might have provided a selection bias. Fourth, coronary angiography was performed only in the minority of cases and no noninvasive verification of coronary status was repeated at the time of inclusion to this study; therefore, we cannot definitely exclude the impact of ischemic heart disease on our results. Fifth, despite the absence of significant contribution from AVF in multivariable models, it is likely that the AVF-related volume overload, more frequent in the HD subset, might have contributed to the between-group differences in cardiac function and morphology [30].

## 5. Conclusions

Preemptive KTx is associated with a better post-transplant cardiac functional and morphological status than the approach to kidney transplantation based on chronic dialysis. This finding may have important prognostic implications for KTx recipients, thus further supporting the choice of preemptive KTx as the preferred strategy.

## Figures and Tables

**Table 1:** Demographic, clinical, and laboratory characteristics of the studied population.

Parameter	Preemptive KTx *n* = 21	HD KTx *n* = 21	*p* Value
age, years (SD)	45.1(12.3)	45.1 (12.1)	0.99
sex, male, *n* (%)	14 (67)	14(67)	1.0
BMI, kg/m^2^ (SD)	23.9 (2.2)	25.2 (3.5)	0.19
time after KTx, months (IQR)	39 (16–60)	41(24–85)	0.72
causes of kidney failure			
glomerulonephritis, *n* (%)	11 (52)	9 (43)	0.75
interstitial nephropathy, *n* (%)	0 (0)	3 (14)	0.23
hypertensive nephropathy, *n* (%)	1 (5)	2 (9)	0.99
diabetic nephropathy, *n* (%)	0 (0)	1(5)	0.99
polycystic kidney disease, n (%)	4 (19)	4 (19)	0.99
other, *n* (%)	6 (28)	2 (9)	0.24
arterio-venous fistula, *n* (%)	3 (14)	14 (67)	0.002
living donor transplantation, *n* (%)	9 (43)	3 (14)	0.09
diabetes mellitus, *n* (%)	1 (5)	5 (23)	0.19
hypertension, *n* (%)	16 (76)	17 (81)	0.71
hemoglobin, g% (SD)	13.7 (2.6)	13.9 (2.2)	0.79
serum creatinine, mg% (SD)	1.54 (0.72)	1.71 (0.86)	0.49
eGFR, mL/min/1.73 m^2^ (SD)	54.0 (15.7)	51.4 (20.5)	0.64
systolic blood pressure, mmHg (SD)	134.3 (15.6)	138.1 (18.4)	0.47
diastolic blood pressure, mmHg (SD)	84.9(10.6)	84.8 (9.3)	0.98
immunosuppressive treatment, n (%)			
tacrolimus, mycophenolate mofetil, steroids	15 (71)	16 (76)	0.99
cyclosporine, mycophenolate mofetil	2 (10)	2 (10)	0.99
tacrolimus, mycophenolate mofetil	2 (10)	3 (14)	0.99
tacrolimus, steroids	1 (5)	0 (0)	0.99
Azatopiryne	0 (0)	1 (5)	0.99
hypotensive treatment, *n* (%)			
beta-blockers	11 (52)	15 (71)	0.34
ACEIs/ARBs	2 (10)	5 (24)	0.41
calcium blockers	8 (38)	10 (48)	0.76
alfa-1 blockers	2 (10)	6 (29)	0.24

A *p*-value of less than 0.05 was considered significant. ACEI, angiotensin-converting enzyme inhibitor; ARB, angiotensin receptor blocker; BMI, body mass index; eGFR, estimated glomerular filtration rate; HD hemodialysis; KTx, kidney transplant.

**Table 2:** Echocardiographic characteristics of the studied population.

Parameter	Preemptive KTx *n* = 21	HD KTx *n* = 21	*p* Value
LV end-diastolic dimension, mm (SD)	47.1 (4.7)	49.3 (3.9)	0.09
Relative wall thickness (SD)	0.43 (0.04)	0.42 (0.05)	0.88
LVMI, g/m^2^ (SD)	94.4 (18.8)	109.8 (18.8)	0.01
Left ventricular hypertrophy, n (%)	4 (19)	12 (57)	0.03
LAVI, mL/m^2^ (SD)	29.4 (4.5)	34.5 (5.3)	0.002
LAVI >34 mL/m^2^, *n* (%)	0 (0)	11 (52)	<0.001
PALS, % (SD)	34.1 (4.7)	25.1 (6.9)	<0.001
PACS, % (SD)	15.9 (1.5)	10.5 (3.8)	<0.001
LV ejection fraction, % (SD)	60.9 (2.3)	62.6 (3.9)	0.10
E/A (SD)	1.07 (0.28)	0.98 (0.28)	0.31
Deceleration time of E wave, ms (SD)	17.2 (37.1)	178.4 (47.2)	0.67
e’ septal, cm/s (SD)	8.0 (1.9)	6.5 (1.7)	0.01
e’ lateral, cm/s (SD)	11.8 (2.3)	8.7 (2.7)	<0.001
E/e’ (SD)	6.9 (1.0)	9.1 (1.4)	<0.001
GLS, % (SD)	20.0 (2.3)	17.1 (3.0)	0.001
basal RV dimension, mm (SD)	32.0 (3.3)	34.1 (3.1)	0.21
TAPSE, mm (SD)	21.5 (2.3)	21.4(1.8)	0.82
RVGLS% (SD)	24.5 (4.8)	20.7 (5.9)	0.03

A *p*-value of less than 0.05 was considered significant. A, peak late diastolic velocity; E, peak early diastolic inflow velocity; e’, peak early diastolic mitral annular velocity; GLS, global longitudinal strain; LAVI, left atrial volume index; LV, left ventricle; LVMI, left ventricular mass index; RV, right ventricle; PACS, peak atrial contractile strain; PALS, peak atrial longitudinal strain; TAPSE, tricuspid annular plane systolic excursion; RVGLS, right ventricular global longitudinal strain.

**Table 3:** Associations of cardiac functional and morphological parameters with demographic and clinical characteristics: univariate analysis.

	Age		Male Sex		BMI		HT		DM		eGFR		HD		AVF	
	R	*p*	R	p	R	*p*	R	*p*	R	*p*	R	*p*	R	*p*	*R*	*p*
LVMI	0.33	**0.03**	−0.35	**0.02**	0.42	**0.005**	0.42	**0.005**	0.14	0.36	−0.31	**0.049**	0.37	**0.02**	0.29	0.06
E/e`	0.08	0.61	0.28	0.07	0.12	0.48	0.17	0.27	0.05	0.76	−0.39	**0.01**	0.69	**<0.001**	0.32	**0.04**
GLS	0.31	**0.048**	0.45	**0.003**	−0.33	**0.03**	−0.10	0.53	−0.29	0.07	0.09	0.51	−0.46	**0.001**	−0.28	0.07
LAVI	0.53	**0.02**	−0.23	0.14	0.02	0.87	0.18	0.25	0.14	0.38	−0.21	0.17	0.47	**0.002**	0.42	**0.005**
PALS	−0.10	0.53	0.06	0.69	−0.29	0.06	−0.23	0.15	−0.31	**0.04**	0.16	0.31	−0.64	**<0.001**	−0.20	0.20
PACS	0.21	0.18	−0.16	0.31	−0.13	0.41	−0.19	0.22	−0.16	0.30	0.19	0.20	−0.67	**<0.001**	−0.34	**0.02**
RVGLS	−0.11	0.51	0.24	0.14	−0.35	**0.03**	−0.14	0.39	−0.01	0.93	−0.11	0.51	−0.33	**0.04**	0.16	0.34

A *p*-value of less than 0.05 was considered significant. AVF, arteriovenous fistula, BMI, body mass index; DM, diabetes mellitus; E, peak early diastolic inflow velocity; e‘, peak early diastolic mitral annular velocity; eGFR, estimated glomerular filtration rate; GLS, global longitudinal strain; HD, hemodialysis; HT, hypertension; LAVI, left atrial volume index; LVMI, left ventricular mass index; PACS, peak atrial contractile strain; PALS, peak atrial longitudinal strain; RVGLS, right ventricular global longitudinal strain.

**Table 4:** Associations of cardiac function and morphological parameters with demographic and clinical characteristics: multivariate analysis.

	Model for LVMI			Model for LAVI			Model for E/e’			Model for GLS			Model for PALS			Model for PACS			Model for RVGLS		
	R^2^ = 0.61			R^2^ = 0.67			R^2^ = 0.60			R^2^ = 0.51			R^2^ = 0.41			R^2^ = 0.50			R^2^ = 0.22		
	β	SE	p	β	SE	p	β	SE	p	β	SE	p	β	SE	p	β	SE	p	β	SE	p
age	0.32	0.10	**0.002**	−0.58	0.09	**<0.001**	-	-	-	0.28	0.11	**0.02**	-	-	-	−0.32	0.12	**0.01**	-	-	-
female sex	−0.83	0.10	**<0.001**	−0.51	0.10	**<0.001**	0.28	0.10	**0.02**	0.46	0.11	**<0.001**	-	-	-	−0.20	0.12	0.09	-	-	-
BMI	0.14	0.11	0.21	−0.31	0.10	**0.006**	-	-	-	-	-	-	−0.19	0.13	0.14	-	-	-	−0.30	0.16	0.06
HT	0.25	0.10	**0.02**	-	-	-	0.20	0.10	0.051	-	-	-	−0.13	0.13	0.32	−0.28	0.11	**0.02**	-	-	-
DM	-	-	-	−0.14	0.10	0.16	−0.16	0.11	0.13	−0.21	0.12	0.08	−0.19	0.13	0.14	-	-	-	-	-	-
eGFR	−0.28	0.11	**0.003**	−0.25	0.09	**0.01**	−0.25	0.11	**0.02**	0.23	0.11	0.06	0.12	0.12	0.32	−0.12	0.12	0.29	−0.15	−0.15	0.32
HD	0.32	0.10	**0.003**	0.55	0.10	**<0.001**	0.68	0.10	**<0.001**	−0.41	0.12	**<0.001**	−0.50	0.13	**<0.001**	−0.60	0.11	**<0.001**	−0.29	0.16	0.07

A *p*-value of less than 0.05 was considered significant. BMI, body mass index; BSA, body surface area; DM, diabetes mellitus; E, peak early diastolic inflow velocity; e, peak early diastolic mitral annular velocity; eGFR, estimated glomerular filtration rate; GLS, global longitudinal strain; HD, hemodialysis; HT, atrial hypertension; LAVI, left atrial volume index; LVMI, left ventricular mass index; PACS, peak atrial contractile strain; PALS, peak atrial longitudinal strain; RVGLS, right ventricular global longitudinal strain.

## Data Availability

The data presented in this study are available on request from the corresponding author.
